# Supplementary material for: Bacteria synthesize a bacteriocin within the cell membrane
Source: mBio. 2026 Sep 10;17(10):e01423-26. doi: 10.1128/mbio.01423-26 (PMC13712780; doi:10.1128/mbio.01423-26)
Supplement: Supplementary file 1 [file mbio.01423-26-s0001.pdf]

SUPPLEMENTAL MATERIAL FOR

Bacteria synthesize a bacteriocin within the cell membrane

Fauzia H. Nur[1,2], Ama N. Antwi[1,2], and Seth W. Dickey[1,2,*]

Affiliations

[1]Department of Veterinary Medicine, University of Maryland, College Park, MD, United States of America

[2]Virginia-Maryland College of Veterinary Medicine, College Park, MD, United States of America

*Correspondence to: sdickey@umd.edu

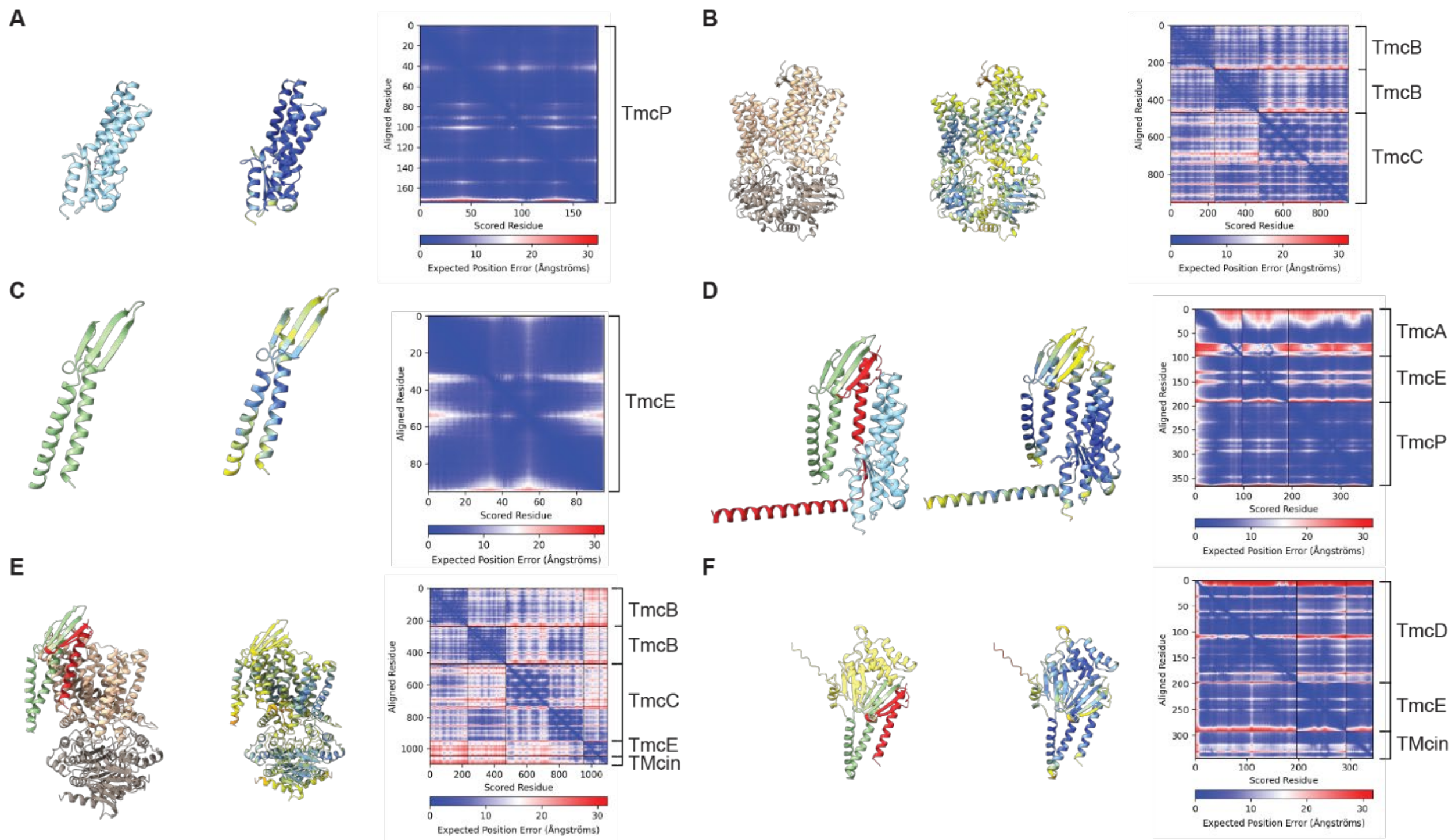

Fig. S1: **Confidence metrics and structures of AlphaFold3 generated models in this study.** (**A-F**) Cartoon representation of proteins and complexes colored by chain (left) and pLDDT score (middle). Predicted aligned error plots are on the right. (**A**) TmcP (one copy, cyan). (**B**) TmcBC with two copies of TmcB ($TmcB_2$, brown) and one copy of TmcC (light brown). (**C**) TmcE (one copy, sage green), (**D**) Complex of TmcE, TmcA (one copy, red), and TmcP. (**E**) Complex of TmcE, TMcin (one copy, red), and $TmcB_2C$. (**F**) Complex of TmcD (one copy, yellow, residues 21-217), TmcE, and TMcin.

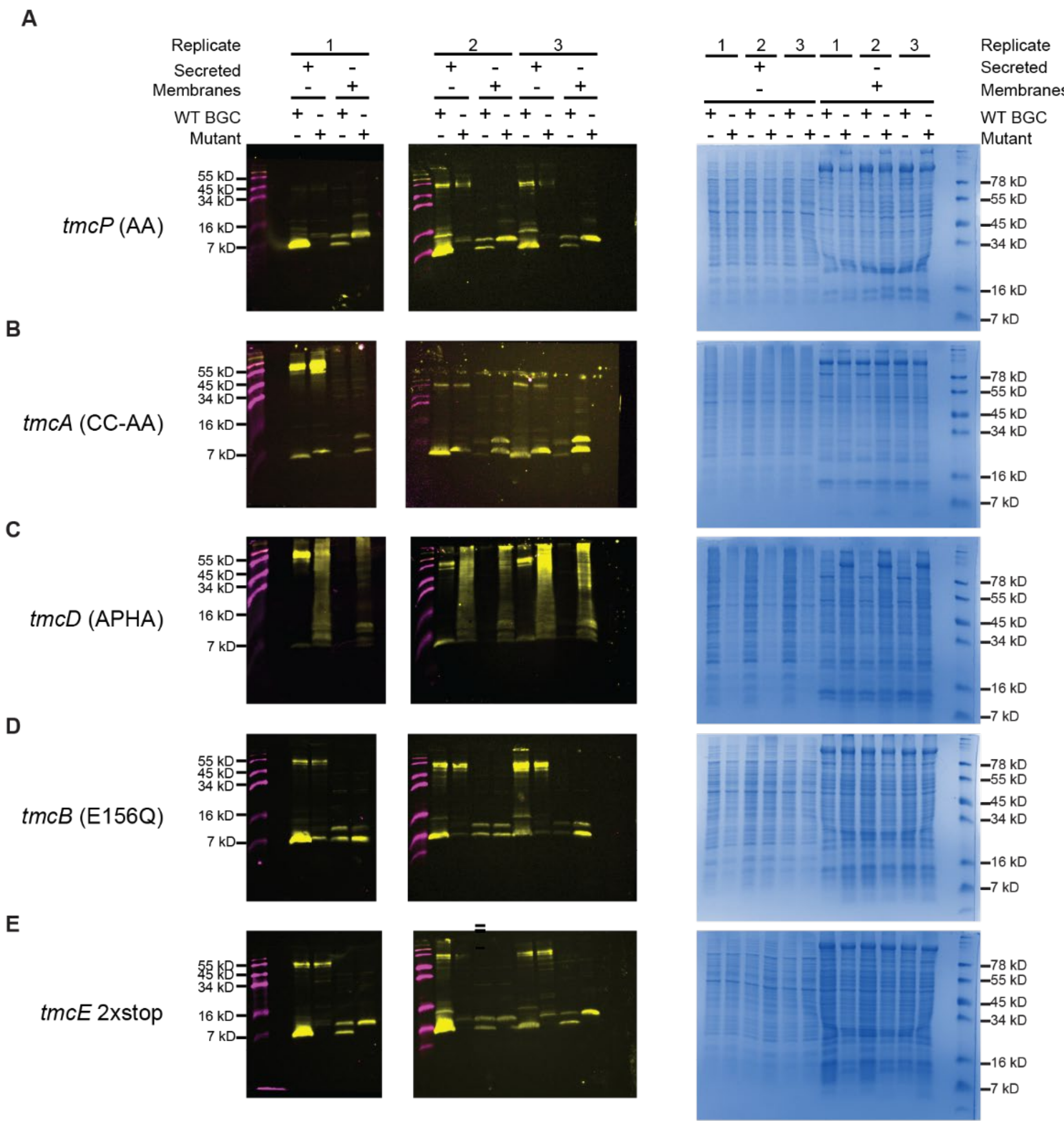

Fig. S2: Replicates of G1905 P_{tet}-TMcinBiosynthesis cells with mutations to individual TMcin BGC genes. Western blots using αTMcin-G1905 (top: yellow, αTMcin; magenta, molecular weight markers) and Coomassie-stained SDS-PAGE gels (bottom) of subcellular fractions after induction of TMcin biosynthesis in cells with mutations to (**A**) *tmcP*, (**B**) *tmcA,* (**C**) *tmcD,* (**D**) *tmcB, and* (**E**) *tmcE.*

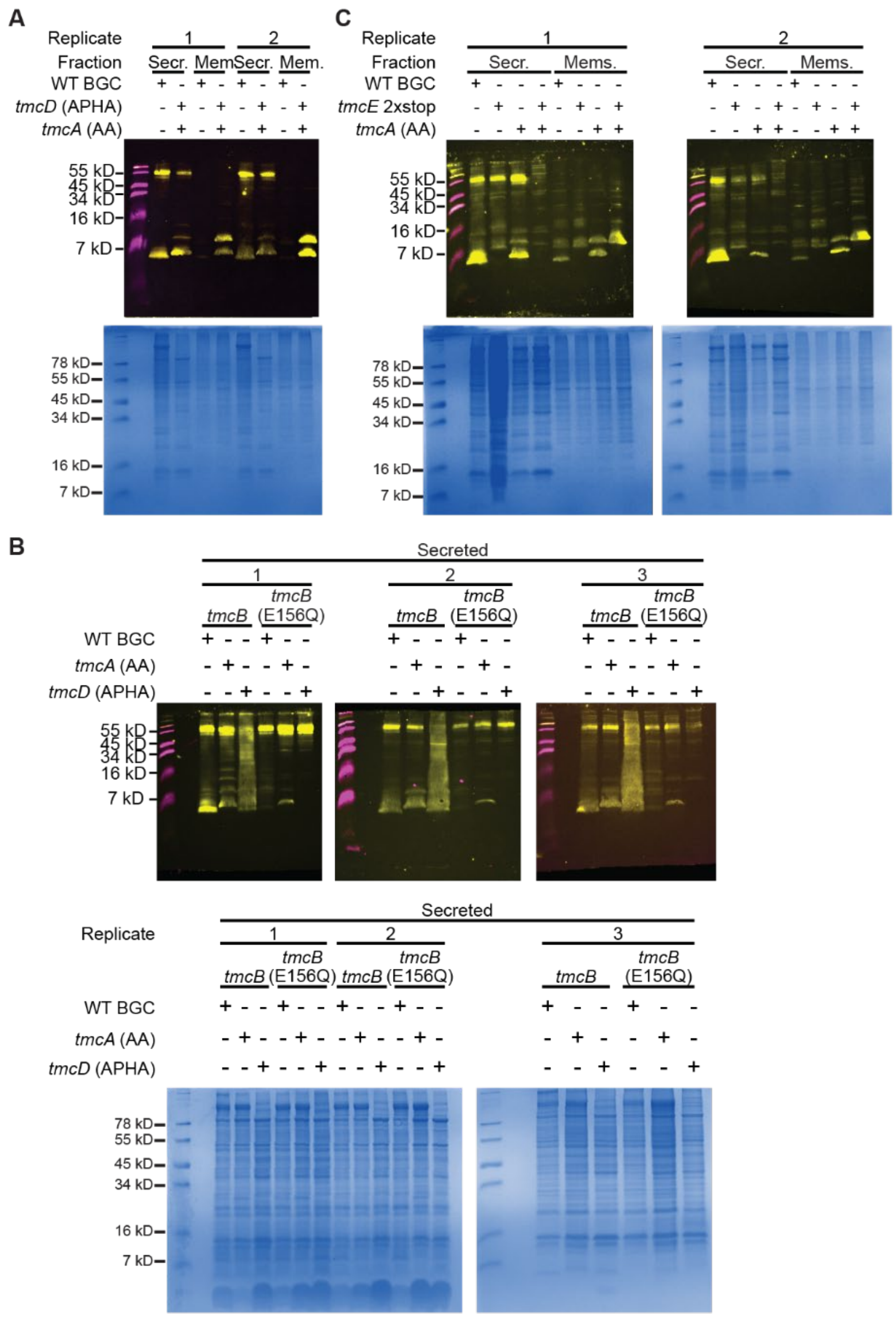

Fig. S3: Replicates of G1905 Ptet-TMcinBiosynthesis cells with mutations to two TMcin BGC genes. Western blots using αTMcin-G1905 (top: yellow, αTMcin; magenta, molecular weight markers) and Coomassie-stained SDS-PAGE gels (bottom) of subcellular fractions after induction of TMcin biosynthesis in cells with mutations to (**A**) *tmcA* and *tmcD*; (**B**) *tmcA* and *tmcB* or *tmcD* and *tmcB*; and (**C**) *tmcA* and *tmcE*.

Table S1: Results from HMMScan using the TmcP sequence as input.

Family id	Family Accession	Clan	Env. Start	Env. End	Model Start	Model End	Bit Score	Ind. E-value	Cond. E-value	Description
DUF3267	PF11667	CL0126	61	171	2	100	26.5	8.50e-06	1.06e-09	Putative zincin peptidase

Table S2: Bacterial strains used in this study

Bacterial Strain	Description	Source
Escherichia coli DC10B	*dcm*⁻ K12 strain used for subcloning pIMAYermC	(1)
S. aureus G1905	Wild-type strain; native producer of TMcin-G1905	(2)
S. aureus G1905 pCT545::pCURE	Mutant strain engineered to lack the TMcin BGC via replacement of pCT545 with the heterologous pCURE plasmid that includes the putative pCT545 TA system	(3)
S. aureus G1905 ΔpCT545 Δ*tetML*	Mutant strain engineered to lack the TMcin BGC via removal of pCT545 without the need for a replacement plasmid; and with engineered deletions of *tetM* and *tetL* to allow for induction using anhydrotetracycline	This study

Table S3: Oligonucleotides used in this study.

Oligonucleotide name	Sequence (5'-3')	Purpose
pCT545_antitoxin_forward	CATATGAGCACAAACCCAAA	Construct pIMAYermC CT545-AT
pCT545_2stop_antitoxin_reverse	TGATAATACTTAATATTTGTTTTGATAATAGCACCG	
TmcA_detect_forward	ATGATGGAAAAAGCAAACCAC	PCR screen for loss of pCT545
TmcA_detect_reverse	AACATCGAGGCTAACCC	
PSMalpha_detect_forward	TGCACCTGTACAGTTAGGCAGT	Positive control amplifying a locus on the chromosome
PSMalpha_detect_reverse	TTGCAACAAACAAGACTCACCT	
IM151	TACATGTCAAGAATAAACTGCCAAAGC	PCR screen for loss of pIMAYermC CT545-AT (1)
IM152	AATACCTGTGACGGAAGATCACTTCG	
1kb_*tetML*_forward	AATCGAATGGAAAGTCTAAAAGG	Construct pIMAYermC Δ*tetML* and PCR screen for Δ*tetML* mutation
*tetML*_1kb_reverse	TTGCAGTTTTCCATCACG	
Δ*tetL*_right	TAAATCGTTAAGGGATCAACTTTG	
Δ*tetM*_left	GTGATTTTCCTCCATTCAAAAAC	
1200_*tetM*_forward	TTGTGATTGCCTTGTGGGTA	
pTX15_MCS_right	ATATGATTTGCGGCCGCATACGCGTGTGATGAACGC TTTATCTTAATGC	Construct pKX17
pTX15_MCS_left	GATATCCTCCTCAGCTGGATCCTACATTTTAGTTGGT TAATTT	
pKX17_ΔPxylA_ApaI_right	TATGCTGGGCCCAGCTGAGGAGGATATCATAT	Construct pKT17, removing *xylR* and P_{xylA}
pKX17_Δ*xylR*_left	GATCATCATGACAGATCCGG	
pIMAY_Pxyl_ApaI_forward	CTGAGGGCCCCTCTATCAATGATAGAGAGCTTATTTT AATTATACTCTATCAATGATAG	Construct pKT17, inserting *tetR*-P_{tet}
pIMAY_*tetR*_reverse	TGAAGTTACCATCACGGA	
*tetR*_remove_NdeI_right	AATTATCAATTCAAGGCCGAATAAG	Construct pKT17, removing extraneous NdeI site
*tetR*_remove_NdeI_left	TGCGGATTAGAAAAACAACTTAAAT	
pRB_ΔGramN_right	GTCATTACCCCAGGCGTTTA	Delete Gram-negative replication and antibiotic resistance marker
pRB_ΔGramN_left	GCATCTGTGCGGTATTTCA	
Δ*tmcA*_right	TAACTTTTAAACTACAAATGCGTGG	Delete *tmcBCEPA* in pRB5gp + BGC
Δ*tmcB*_left	TTGTAAGTCGTCCTTTAATTTTTTTAAATATATATAATA TTG	
*tmcB*_NdeI_forward	TCATGTCATATGTTGAAAATTGAAAATGTATCATTTAA GTATAC	Construct pKT17 BGC-core, inserting *tmcB*CEPA
*tmcA*_NotI_reverse	ATACCAGGCGGCCGCTTATCTTTTGCACTCAACATCG	
*tmcD*_R70_right	AGAACTCTATATAATAATATTATAAAAAAAATTGATGA TGG	*tmcD* APHA mutagenesis
*tmcD*_APHA_left	TGCGTGAGGAGCTTTAAAATCTTCGAAAACTATAATT TTATTTTTTGAAC	
*tmcB*_E156Q_right	CAAACATTAAATGGAATGGATATTGAATCA	*tmcB* E156Q mutagenesis
*tmcB*_D155_left	ATCTATTATTAGAATATTGGGTGCTATTAAC	
*tmcE*_S10L11Stop_right	TAATAGGTTTTTTCTATAGTAACTACCGGAAT	*tmcE* 2xstop mutation
*tmcE*_N9_left	GTTAATTAAATATATTAAGTGTTTCATTTCTCGAC	
*tmcP*_H68A_right	GCAGAATATATGCATATATATTTCTTGAAGAAAAAGC	*tmcP* H68A mutagenesis
*tmcP*_H68A_left	TATAGCAAAAGAAAGAAGCAAACC	
*tmcP*_D158A_right	GCAGGAGTAATGTTAATCAAAGGAATATTAAAG	*tmcP* D158A mutagenesis
*tmcP*_D158A_left	TCCTAATGGTGGAAGTATGTTAA	
*tmcA*_C70A_right	GCAGTAGTGAATCAACATGGTAAGTT	*tmcA* C70A mutagenesis
*tmcA*_C70A_left	CCAAATAGTTAAACCAGCAAAAATT	
*tmcA*_C95A_right	AAAAGATAAGCGGCCGCA	*tmcA* C95A mutagenesis
*tmcA*_C95A_left	TGCCTCAACATCGAGGCTAAC	

Table S4: Plasmids used in this study.

Plasmid Name	Description/Purpose	Source
pIMAYermC	Temperature sensistive plasmid conferring erythromycin resistance	(3)
pIMAYermC CT545-AT	Cure G1905 of plasmid pCT545 without needing a replacement plasmid	This study
pIMAYermC Δ*tetML*	Delete *tetML* to allow for induction by aTc	This study
pKX15	Source to construct pKX17; xylose inducible plasmid	(4)
pKX17	Source to construct pKT17; xylose inducible plasmid	This study
pKT17	aTc-inducible plasmid	This study
pKT17+BGC-core	Inducible TMcin BGC complementation plasmid	This study
pKT17+BGC-core *tmcB*(E156Q)	TmcBC loss of function mutation	This study
pKT17+BGC-core *tmcE*(2xstop)	TmcE loss of function mutation	This study
pKT17+BGC-core *tmcP*(H68A,D158A)	TmcP loss of function mutation	This study
pKT17+BGC-core *tmcA*(C70A,C95A)	TmcA disulfide bond mutation	This study
pKT17+BGC-core *tmcE*(2xstop) *tmcA*(C70A,C95A)	Combination of TmcA disulfide bond and TmcE loss of function mutations	This study
pKT17+BGC-core *tmcB*(E156Q) *tmcA*(C70A,C95A)	Combination of TmcA disulfide bond and TmcBC loss of function mutations	This study
pRB573	*E. coli* and *S. aureus* shuttle plasmid	(5)
pRB5gp	Smaller plasmid replication-competent in staphylococci only	This study
pRB+BGC	TMcin BGC complementation plasmid	(*8*)
pRB+BGC (HA-*tmcA*)	TMcin BGC complementation plasmid with a HA tag fused to the 5' end of *tmcA*	This study
pRB5gp+BGC	TMcin BGC complementation plasmid replication-competent in staphylococci only	This study
pRB5gp+BGC-Δcore	Deletion of the *tmcBCEPA* operon	This study
pRB5gp+BGC-Δcore *tmcD*(C66A,C69A)	TmcD loss of function mutation	This study

REFERENCES

1. Monk IR, Shah IM, Xu M, Tan MW, Foster TJ. 2012. Transforming the untransformable: application of direct transformation to manipulate genetically Staphylococcus aureus and Staphylococcus epidermidis. mBio 3.
2. Amissah NA, Glasner C, Ablordey A, Tetteh CS, Kotey NK, Prah I, van der Werf TS, Rossen JW, van Dijl JM, Stienstra Y. 2015. Genetic diversity of Staphylococcus aureus in Buruli ulcer. PLoS Negl Trop Dis 9:e0003421.
3. Dickey SW, Burgin DJ, Antwi AN, Villaruz A, Galac MR, Cheung GYC, Rostovtseva TK, Worrall LJ, Lazarski AC, Cino EA, Tieleman DP, Bezrukov SM, Strynadka NCJ, Otto M. 2025. Antimicrobial peptide class that forms discrete β-barrel stable pores anchored by transmembrane helices. Nat Commun 16:7231.
4. Gauger T, Weihs F, Mayer S, Krismer B, Liese J, Kull M, Bertram R. 2012. Intracellular monitoring of target protein production in Staphylococcus aureus by peptide tag-induced reporter fluorescence. Microb Biotechnol 5:129–134.
5. Schaaff F, Reipert A, Bierbaum G. 2002. An elevated mutation frequency favors development of vancomycin resistance in Staphylococcus aureus. Antimicrob Agents Chemother 46:3540–3548.
